# Irrefutable evidence for the use of docetaxel in newly diagnosed metastatic prostate cancer: results from the STAMPEDE and CHAARTED trials

**DOI:** 10.1186/s12916-015-0543-9

**Published:** 2015-12-22

**Authors:** Robert J. van Soest, Ronald de Wit

**Affiliations:** Departments of Urology, Erasmus University Medical Center and Cancer Institute, Rotterdam, The Netherlands; Department of Medical Oncology, Erasmus University Medical Center and Cancer Institute, P.O. Box 5201, 3008AE Rotterdam, The Netherlands

**Keywords:** Androgen deprivation therapy, Docetaxel, Metastatic castration-resistant prostate cancer, Metastatic hormone-naïve prostate cancer, Taxanes

## Abstract

Androgen deprivation therapy (ADT) has been used in the treatment of metastatic prostate cancer since the first description of its hormonal dependence in 1941. In 2004, docetaxel chemotherapy became the mainstay of treatment in metastatic castration-resistant prostate cancer (mCRPC), following robust, albeit modest, survival benefit in two randomized phase 3 trials. The recently published CHAARTED trial was the first to show that combining ADT with docetaxel in men with hormone-naïve (hormone-sensitive) metastatic prostate cancer (mHSPC) yielded a remarkable overall survival benefit of 13.6 months as compared with ADT alone. In the current issue of *The Lancet*, James et al. report results of the STAMPEDE trial in men with high-risk locally advanced or metastatic prostate cancer initiating long-term hormone therapy. The combination of six cycles of docetaxel with ADT in men commencing long-term ADT demonstrated a similar OS benefit compared with standard of care (SOC) by a median of 10 months. Based on the consistency of the data and the firmness of the benefit provided, docetaxel in addition to ADT should be considered SOC for men with newly diagnosed mHSPC.

## Background

### Docetaxel in metastatic castration-resistant prostate cancer (mCRPC)

Docetaxel has been available for more than 10 years for the treatment of metastatic castration resistant prostate cancer (mCRPC) [1, 2]. The overall survival (OS) benefit obtained in the pivotal study TAX 327, comparing docetaxel plus prednisone and mitoxantrone plus prednisone, was 2.9 months in the final analysis [3]. Since this survival benefit was obtained despite confounding effects by cross-over to docetaxel in a third of patients failing mitoxantrone, and the benefit in terms of symptom and quality of life improvements during docetaxel treatment is frequently obvious, its use was rapidly adopted. With the advent of the orally-administered novel androgen receptor (AR) targeted agents, abiraterone and enzalutamide, docetaxel is increasingly becoming positioned as second- or even third-line treatment after these AR-targeted treatments. Survival benefit obtained in first-line or second-line settings, however, may be quite different when used in subsequent lines of treatment. This concern is fueled by increasing evidence from both preclinical and clinical studies indicating that the efficacy of docetaxel is impaired when used after novel AR-targeted therapy [4–6]. Using docetaxel early in the treatment of prostate cancer, prior to these agents, might thus provide a new treatment opportunity in the management of metastatic prostate cancer.

### The use of docetaxel in hormone-naïve metastatic prostate cancer (mHSPC)

Androgen deprivation therapy (ADT) has been used in the treatment of metastatic prostate cancer since the first description of its hormonal dependence in 1941 [1]. Although it is the gold standard treatment for metastatic prostate cancer, disease progression ultimately becomes inevitable. The hypothesis that a subpopulation of prostate cancer cells may be AR-negative, and thus resistant to ADT from the beginning, was the rationale for combining ADT with docetaxel in men with hormone-naïve metastatic prostate cancer (mHSPC). In the CHAARTED study [7], 790 men with mHSPC were randomized to six cycles of docetaxel plus ADT versus ADT alone. Men were stratified according to high- or low-volume disease, with high-volume defined as visceral metastases or ≥4 bone lesions with ≥1 beyond the vertebral bodies and pelvis. Median OS was 57.6 months for men treated with docetaxel plus ADT versus 44 months for ADT alone (13.6 months longer, hazard ratio (HR), 0.61; 95 % confidence interval (CI), 0.47–0.80; *P* <0.001). In men with high-volume disease, the treatment effect of docetaxel plus ADT was even more pronounced, with an OS benefit of 17 months (49 vs. 32 months; HR, 0.60; 95 % CI, 0.45–0.81; *P* <0.001). In the low-volume subgroup, median OS was not yet reached.

STAMPEDE is a multi-arm, multistage trial that included both metastatic and non-metastatic prostate cancer patients [8]. The current report of the STAMPEDE trial reflects results of four study arms, including standard of care (SOC; ADT ± radiotherapy), SOC plus docetaxel, SOC plus zoledronic acid, and SOC plus docetaxel and zoledronic acid [8]. Docetaxel was administered as six cycles of 75 mg/m^2^ every 3 weeks with prednisone 10 mg daily. Approximately 61 % of patients had metastatic disease (by TNM criteria) at the time of inclusion in the study, whereas 15 % presented with node positive disease and 24 % with high-risk locally advanced disease (T3/4, PSA >40 or Gleason 8–10). The STAMPEDE study demonstrated that docetaxel in combination with SOC improved OS by 10 months as compared with SOC alone (HR, 0.78; 95 % CI, 0.66–0.93; *P* = 0.006). When confined to patients with metastatic disease, the OS benefit was 15 months for docetaxel plus SOC versus SOC alone (HR, 0.76; 95 % CI, 0.62–0.92; *P* = 0.005). For patients with non-metastatic disease, median OS was not yet reached and statistical significance could not be demonstrated.

## Findings in perspective

Two large randomized trials have now shown a robust and clinically meaningful survival benefit by the addition of docetaxel to ADT in men with mHSPC [7, 8]. The magnitude of the OS in the CHAARTED and STAMPEDE trials is remarkable (13.6 and 15 months in the metastatic patient population, respectively) and much larger than obtained with the use of chemotherapeutic or novel hormonal agents in the setting of mCRPC. A meta-analysis of the available data, also incorporating a smaller study by the French Genitourinary Tumor Group (GETUG-15) that showed a similar trend but did not reach statistical significance, is reported by Vale et al. [9] in the current issue of *The Lancet Oncology*. The meta-analysis revealed a robust 9 % absolute OS benefit at 4 years by the use of docetaxel plus ADT in men with mHSPC [9]. Consequently, these results should be considered as practice changing in the daily treatment of men with prostate cancer. Men with newly diagnosed mHSPC, who are considered fit to receive chemotherapy, should be offered six cycles of docetaxel in addition to ADT.

However, some questions remain unsolved. Which patients precisely benefit from six cycles of docetaxel? Should docetaxel be administered with or without prednisone? And what is the explanation for the magnitude of the benefit by applying docetaxel early?

The CHAARTED study stratified for high- versus low-volume disease and only showed a statistically significant OS benefit in the high-volume subgroup. For men with low-volume disease, the number of events was too small at the time of reporting and therefore longer follow-up is awaited. The STAMPEDE trial, on the other hand, did not stratify for volume of metastatic disease and demonstrated clinical benefit for the entire patient population. These findings, in by far the largest study, in combination with the results from the meta-analysis incorporating low-volume metastatic patients, demonstrate that docetaxel plus ADT should be considered in all men with mHSPC regardless of disease burden.

Whereas the benefit of docetaxel is clearly visible in metastatic patients, longer follow-up will be needed to draw conclusions for non-metastatic patients. Likewise, since the vast majority of men included in both STAMPEDE and CHAARTED studies presented with newly diagnosed metastatic prostate cancer, conclusions regarding the use of docetaxel in men previously treated for local disease are also limited. To date, the recommendation of docetaxel plus ADT thus reflects the setting of newly diagnosed metastatic disease. For those patients, however, in whom metastatic disease becomes apparent within months after local treatment and the true extent of disease may have been missed upfront, treatment decisions should be individualized and preferably weighed in the multidisciplinary setting of modern patient care.

The reasons that underlie the greater benefit by using six cycles of docetaxel in the hormone-naïve setting as compared to the castrate-resistant setting might be sought in the early kill of hormone-resistant cell clones, as previously suggested by Sweeney et al. [7]. An alternative or additional explanation could lie in different docetaxel pharmacokinetics. The incidence of neutropenic fever in TAX 327 was 2.7 %, whereas the incidence in all three mHSPC studies ranged from 6 to 12 %. These findings may suggest different docetaxel exposure in mCRPC as compared with mHSPC patients. Franke et al. [10] have shown that the clearance of docetaxel is affected by a castrate status. In their study, the clearance of docetaxel was increased by 100 % in castrated as compared to non-castrated men, resulting in a significantly higher area under the curve.

Steroids are CYP3A4 inducers and may therefore influence taxane pharmacokinetics. A recent Danish report suggested a higher incidence of neutropenic fever in patients receiving docetaxel alone as compared to docetaxel with prednisone [11]. In the CHAARTED trial, docetaxel was given without prednisone. However, STAMPEDE produced similar survival benefit as observed in CHAARTED despite docetaxel being co-administered with prednisone. Although it is conceivable that prednisone might impact docetaxel clearance and may reduce docetaxel exposure, the addition of prednisone clearly enhances the efficacy of the regimen. To substantiate, the recently reported study on orteronel plus prednisone versus prednisone alone in chemotherapy-naïve men with mCRPC showed a 28 % prostate-specific antigen (PSA) response rate and 25 % circulating tumor cell conversion rate at 12 weeks in men receiving prednisone alone [12]. Considering these data, the recommendation is to co-administer docetaxel with prednisone.

## Conclusions

In conclusion, the survival benefit obtained by docetaxel in men with mHSPC is consistent, and much larger than when given at the time of mCRPC. Thus, six cycles of docetaxel plus ADT should be the new standard of care in men with newly diagnosed metastatic prostate cancer.

## Abbreviations

ADT: Androgen deprivation therapy
mCRPC: Metastatic castration-resistant prostate cancer
mHSPC: Metastatic hormone-naïve prostate cancer
SOC: Standard of care
